# A Novel Approach to the Design of a Solid Bismuth Microelectrode Array: Applications in the Anodic Stripping Voltammetry of Cd(II) and Pb(II)

**DOI:** 10.3390/molecules30132743

**Published:** 2025-06-26

**Authors:** Mieczyslaw Korolczuk, Iwona Gęca, Paulina Mrózek

**Affiliations:** Institute of Chemical Sciences, Faculty of Chemistry, Maria Curie Sklodowska University, 20-031 Lublin, Poland; mieczyslaw.korolczuk@mail.umcs.pl (M.K.); paulina.mrozek01@wp.pl (P.M.)

**Keywords:** microelectrode array, anodic stripping voltammetry, heavy metals, determination, environmental monitoring

## Abstract

A new type of solid bismuth microelectrode array characterized by eco-friendly properties and the simplicity of its construction is presented for the first time. The proposed array of microelectrodes consists of exactly forty-three single capillaries of an inner diameter of about 10 µm filled with metallic bismuth and packed in one casing. The proposed sensor is reusable thanks to its distinctive design. The microelectrode properties of the proposed working electrodes were confirmed by comparing the analytical signals of cadmium and lead recorded from stirred and unstirred solutions during the deposition step. The practical application of the solid bismuth microelectrode array is presented by detailing the procedure for the simultaneous determination of Pb and Cd by anodic stripping voltammetry. The calibration graphs were linear from 5 × 10^−9^ to 2 × 10^−7^ mol L^−1^ and 2 × 10^−9^ to 2 × 10^−7^ mol L^−1^ for Cd(II) and Pb(II), respectively (deposition time of 60 s). The detection limits for Cd(II) and Pb(II) were equal to 2.3 × 10^−9^ mol L^−1^ and 8.9 × 10^−10^ mol L^−1^, respectively. Potential interferences were investigated. The developed procedure was successfully used for the analysis of certified water reference material and environmental water samples.

## 1. Introduction

The environmental monitoring of heavy metals is of great importance because of their high toxicity even at low concentration levels, their non-biodegradability, and their ability to accumulate in the tissues of living organisms. As a consequence, the determination of, e.g., Pb(II) and Cd(II) contents in the environmental samples has attracted a significant and common interest.

A variety of analytical methods have been employed for the purpose of quantifying the content of Pb(II) and Cd(II) ions. To date, the following methods have been reported: atomic absorption spectrometry (AAS) [1,2], inductively coupled plasma optical emission spectrometry (ICP-OES) [3,4], inductively coupled plasma mass spectrometry (ICP-MS) [5,6], atomic fluorescence spectrometry (AFS) [7], X-ray fluorescence [8], and high-performance liquid chromatography (HPLC) [9,10,11]. Stripping voltammetry can be used as an alternative to the aforementioned analytical methods on account of the relatively modest cost of the apparatus, portable equipment, and relatively short measurement times. Furthermore, due to the preconcentration step included in the measurement procedure, it can be achieved with very low, comparable, or even competitive detection limits. The comparison of the various analytical methods has been previously reported [12].

In the course of stripping voltammetric measurements, the selection of the working electrode is of great importance. The possibility of electroanalytical determinations within a specified solution depends on the physical and chemical properties of the electrode material, the characteristics of the electrode’s surface or the film plated on its surface, and the operational range of potential values of the given electrode. It is further acknowledged that the quality of analytical results, e.g., the detection limit or the separation of the analytical signals, is influenced by the type of working electrode employed for the measurements. The possible application of the working electrodes in flow conditions is also within the scope of interest of scientists [13], with the advantage of complementing static methods.

Because mercury, in former times commonly used as an electrode material, is highly toxic, intensive research has been undertaken to identify less toxic and more environmentally friendly materials for the preparation of working electrodes. Given the current trend of replacing toxic mercury electrodes with other electrode materials that are considered to be better environmental choices, a range of metal film electrodes made of, e.g., gold [14], bismuth [15], or copper [16,17] have been developed for application in the field. The disadvantage of this solution is the need for the addition of metal ions, e.g., Bi^3+^, to the supporting electrolytes, which, in turn, is associated with the generation of toxic waste.

Another solution is the application of solid metal electrodes, e.g., lead [18] or bismuth [19] electrodes, for voltammetric measurements, where the metallic bismuth/lead constitutes the electrode material. The adoption of this design solution has the advantageous consequence of eliminating the metal film generation step, thereby simplifying and shortening the measurement procedure and eliminating the production of toxic effluents.

In recent times, microelectrodes defined in [20] have attracted considerable attention from electrochemists as a potential alternative to working electrodes of conventional sizes. This is due to the following issues. It is reported that the presence of spherical diffusion at the microelectrode surface leads to the possibility of executing measurements from unmixed solutions and, consequently, to the potential simplification of the measurement procedure and the chance to conduct measurements under field conditions. Next, there is the possibility of analyzing very small sample volumes, including solutions with a low concentration of supporting electrolyte and solutions of organic solvents. Moreover, it has been demonstrated that a favorable signal-to-noise ratio can be achieved when employing working microelectrodes in comparison to conventionally sized electrodes [21,22].

A particular group of microelectrodes are solid metal microelectrodes, which, thanks to their specific design, result in simplified supporting electrolyte composition and the reduced production of toxic wastes. Additionally, they present an alternative to the aforementioned [18,19] solid metallic electrodes, given that a significantly smaller amount of metal is required for their construction. Metal microelectrodes have been constructed in two different forms: as individual (single) microelectrodes and as arrays of microelectrodes [23,24,25,26].

The interest from researchers in the topic of determining Cd and Pb ions using microelectrodes is evidenced by the large number of scientific articles on the subject. Cd and Pb ions were reported to be determined by single microelectrodes and arrays of microelectrodes, which exhibit a variety of design options such as hemispherical mercury microelectrodes [27], on-chip-generated mercury microelectrodes [28], a vibrating silver amalgam microwire electrode [29], bare gold-disk microelectrodes [30], bismuth-coated carbon fiber microelectrodes [31,32], a Hg-electroplated-Ir microelectrode array [33], a gel-integrated microelectrode array [34], a nitrogen-doped diamond-like carbon microelectrode array [35], a mercury-plated diamond-like carbon microelectrode array [36], a micro-sensor based on layer-by-layer self-assembled graphene and bismuth nanoparticles [37], and others [38,39,40,41,42].

In this article, a novel type of solid bismuth microelectrode array with a new and simple design is presented. The important advantage of the proposed working electrode is the fact that the surface of the microelectrode does not have to be modified with bismuth, and, as a consequence, the developed analytical procedure is simplified, shortened, and eco-friendly. The presented solid bismuth microelectrode array was used for the development of a voltammetric procedure for the simultaneous determination of Cd(II) and Pb(II). Satisfactory results for the analysis of water-certified reference material and environmental water samples confirmed the correctness of the presented procedure.

## 2. Results

The new type of working electrode for voltammetric measurements—a solid bismuth microelectrode array—is presented in this article. Many important advantages characterize the presented sensor, such as the simplicity of its construction, environmentally friendly properties, and the possibility of its long-term use. The examination of the microelectrode properties of the presented working electrode and the application of the sensor to determine inorganic ions via anodic stripping voltammetry are described below.

### 2.1. The Examination of Microelectrode Properties

**Figure 1:**
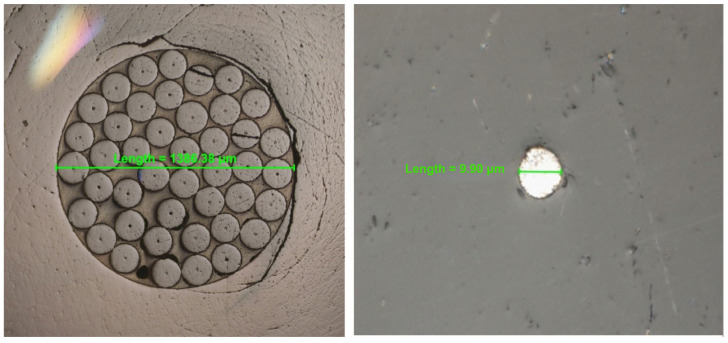
A real image of the surface of the solid bismuth microelectrode array presented in two different close-up images. The images were taken by an inverted metallographic microscope.

**Figure 2:**
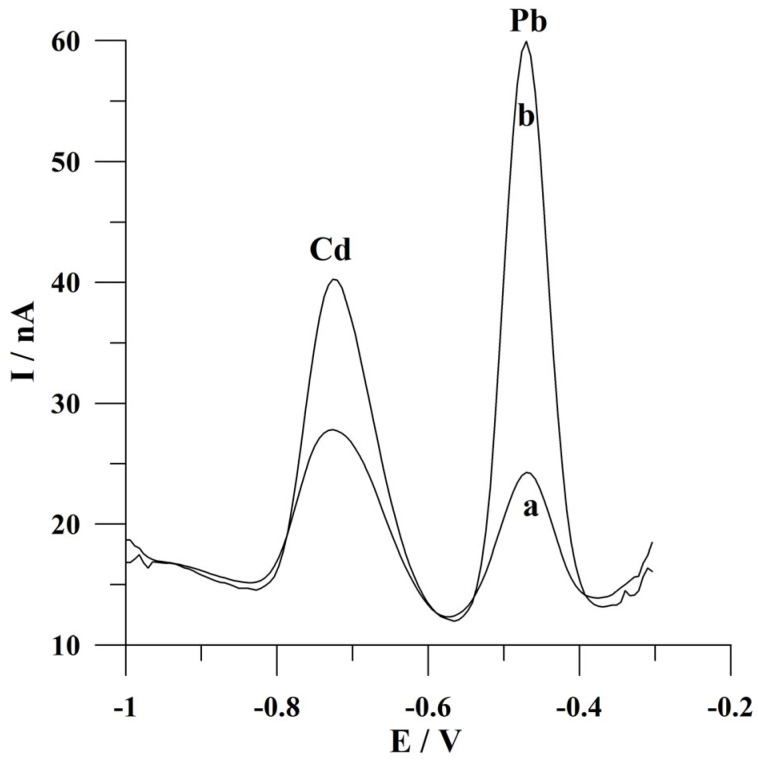
Voltammograms obtained for cadmium and lead determination from (a) an unmixed solution during the deposition step; (b) a mixed solution during the deposition step. The potential for and time of deposition: −0.9 V and 60 s. The concentration of Cd(II) and Pb(II) was 2 × 10^−7^ mol L^−1^. The supporting electrolyte was a 0.05 mol L^−1^ acetate buffer (pH 4.6).

The real image of the surface of the solid bismuth microelectrode array taken by an inverted metallographic microscope is shown in Figure 1. To verify the presence of microelectrode properties of the presented working electrode, the analytical signals of cadmium and lead were recorded from mixed and unmixed solutions during the deposition step. The results obtained are shown in Figure 2. As can be seen, the peak currents of cadmium and lead are about two and five times lower than the unmixed solution, respectively, in comparison to the mixing conditions of the solution when a solid bismuth microelectrode array is used. The reason for this observation is the fact that near the surface of the proposed working microelectrode, mass transport is mainly carried out through the process of spherical diffusion, which is also indicative of the presence of microelectrode properties. This represents a significant advantage of microelectrodes over electrodes of conventional dimensions. In the latter electrodes, the observed peak current obtained under the conditions of an unstirred solution during the deposition stage was several times smaller compared to the signal heights obtained from mixed solutions [26]. This fact makes microelectrodes an interesting and alternative tool for fieldwork.

It is evident from data in the extant literature [20] that currents recorded using the arrays of microelectrodes are amplified and more resistant to noise interferences. To prove this fact, comparative research was carried out using a single solid bismuth microelectrode (d 25 μm) and the solid bismuth microelectrode array presented in this article. The recorded peaks’ currents were higher when the solid bismuth microelectrode array was used. For the case under discussion, an approximate nine-fold amplification of the Cd analytical signal and an approximate five-fold amplification of the Pb analytical signal were obtained when using the presented solid bismuth microelectrode array. In addition, a comparison of background currents showed the enhancement of recorded currents when the solid bismuth microelectrode array was used, which is another advantage of using this type of working electrode.

### 2.2. Influence of Acetate Buffer Concentration on Cadmium and Lead Peak Currents

The supporting electrolyte—an acetate buffer with a pH of 4.6—was chosen based on data from the literature [31,35]. Measurements to select the optimal concentration of acetic buffer were performed in a solution containing 5 × 10^−8^ mol L^−1^ of Pb(II) and Cd(II). The concentration of acetate buffer was changed from 0.025 to 0.25 mol L^−1^. It was observed that the peak currents of both analytes attained the highest value at an acetate buffer concentration of 0.05 mol L^−1^ and decreased at higher concentrations of the supporting electrolyte. As a result of these investigations, a concentration for the acetate buffer of 0.05 mol L^−1^ was chosen for further studies.

### 2.3. The Influence of Conditions for Activation Steps on Cadmium and Lead Peak Currents

**Figure 3:**
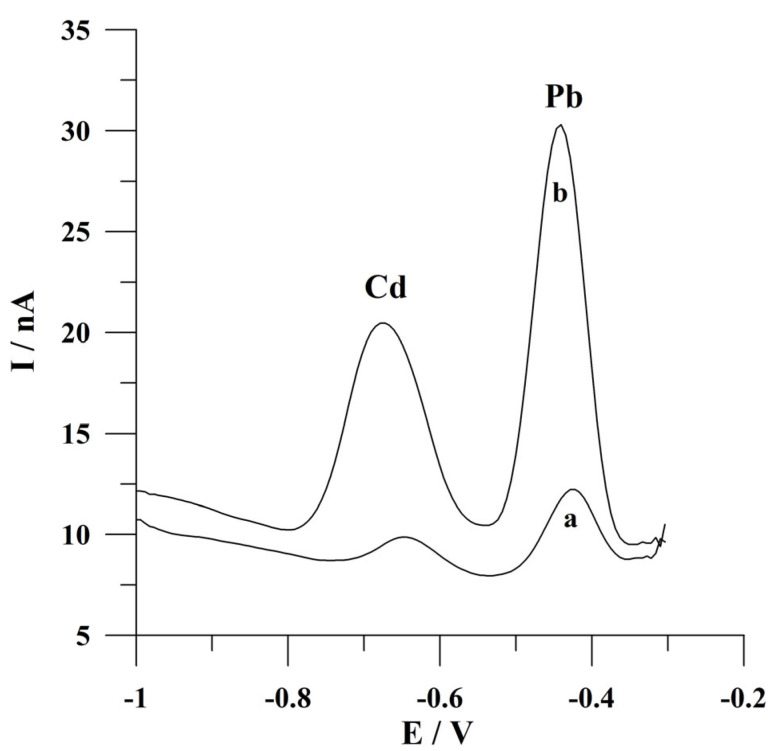
The anodic stripping voltammograms of Cd(II) and Pb(II) obtained when the activation step was (a) not applied and (b) applied to the solid bismuth microelectrode array. The concentrations of Cd(II) and Pb(II): 1 × 10^−7^ mol L^−1^ and 5 × 10^−8^ mol L^−1^, respectively. Deposition conditions: −0.9 V and 120 s. Activation conditions: −2.5 V and 2 s. The supporting electrolyte was a 0.05 mol L^−1^ acetate buffer (pH 4.6).

The so-called activation is a crucial step when using solid metal electrodes. This step is introduced at the beginning of voltammetric measurements and involves the application of a brief, high-negative-potential pulse to the working electrode for a period of a few seconds. As previously reported [26], during this stage of the measurement, the surface of the working electrode is prepared before a proper voltammetric measurement by reducing metal oxides that can be formed as a result of the oxidation of the electrode material with oxygen dissolved in the analyzed solution. The significance of incorporating this step into the voltammetric measurement of the analytical procedure under development is illustrated in Figure 3.

**Figure 4:**
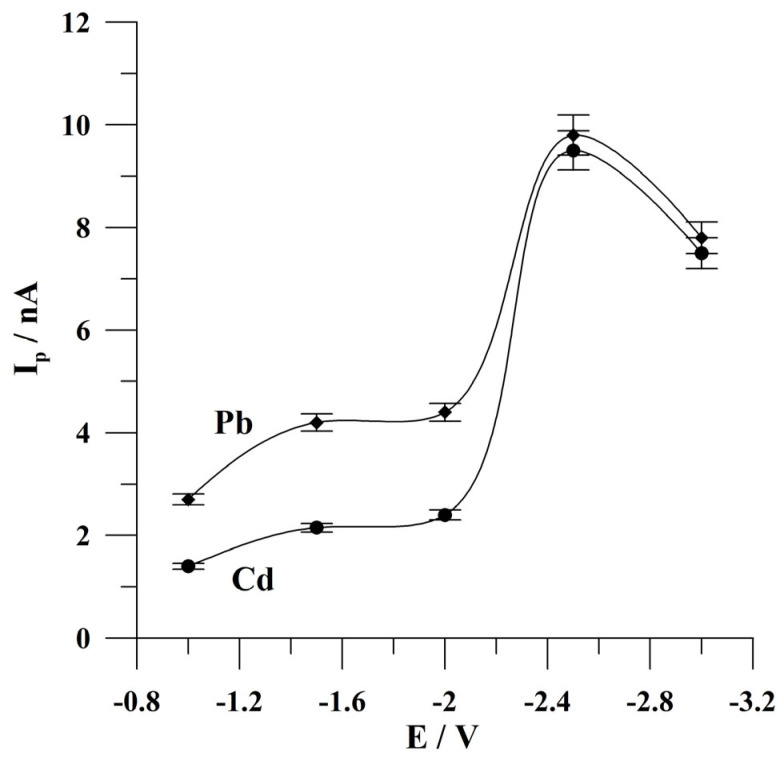
The effect of the activation potential on the peak currents of Cd and Pb. The concentrations of Cd(II) and Pb(II): 1 × 10^−7^ mol L^−1^ and 5 × 10^−8^ mol L^−1^, respectively. Deposition conditions: −0.9 V and 120 s. The error bars refer to the standard deviation (n = 3). The supporting electrolyte was a 0.05 mol L^−1^ acetate buffer (pH 4.6).

Firstly, the potential of the activation step was optimized. During this study, the activation potential was changed from −1.0 to −3.0 V. The concentrations of Cd(II) and Pb(II) were 1 × 10^−7^ mol L^−1^ and 5 × 10^−8^ mol L^−1^, respectively. The results obtained are presented in Figure 4. It was observed that the peak currents of both ions of interest increased from −1.0 V to −2.5 V, attained the highest peak currents at −2.5 V, and then decreased to more negative potential values. The observed decrease is probably connected with the intensive production of hydrogen bubbles, which partially block the surface of the microelectrode. For further studies, the activation potential of −2.5 V was chosen.

Then, the activation time was studied. During this research, the activation time was changed from 0 to 5 s. The concentrations of Cd(II) and Pb(II) were 1 × 10^−7^ mol L^−1^ and 5 × 10^−8^ mol L^−1^, respectively. It was observed that the peak currents of both analyzed ions increased significantly when even a short activation step was applied in the standard procedure of the measurement. It was observed that a one-second potential pulse at a potential of −2.5 V increased the cadmium and lead peaks by approximately 6.7 and 5 times, respectively, compared to measurements without the application of the activation step. Furthermore, it was found that peak currents of cadmium and lead attained the highest values at an activation time of 1 s and then decreased to about 50% of these values at longer activation times. The reason for such an observation can be explained by the reduction in hydrogen ions, which occurs under such conditions. However, based on data in the literature [43], the analytical signals obtained after deposition conducted at highly negative potential values are of high reproducibility when using microelectrodes as the working electrodes. For further studies, an activation time of 1 s was selected as an optimal value.

### 2.4. The Influence of Deposition Conditions on Cadmium and Lead Peak Currents

**Figure 5:**
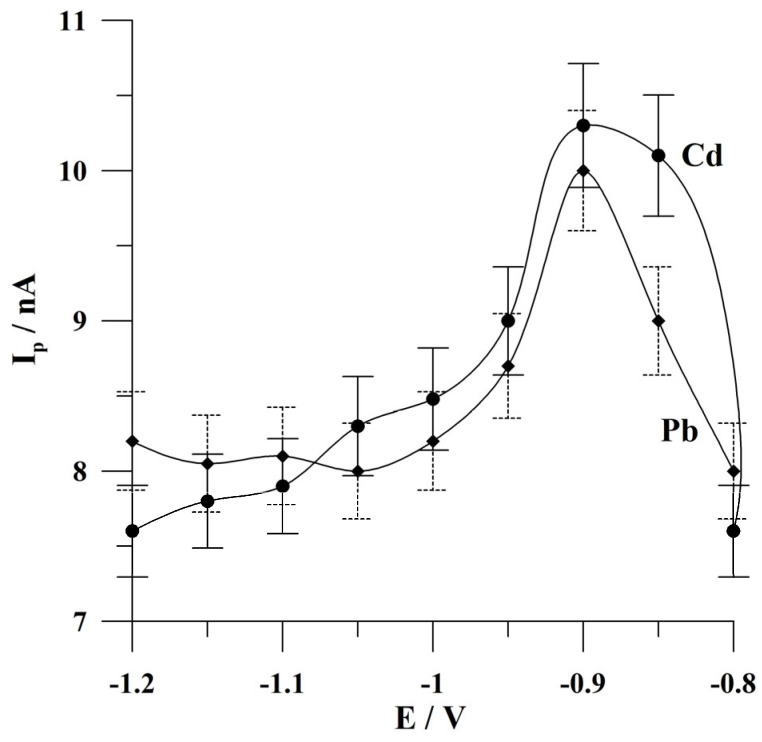
The effect of deposition potential on the peak currents of Cd and Pb. The concentrations of Cd(II) and Pb(II): 1 × 10^−7^ mol L^−1^ and 5 × 10^−8^ mol L^−1^, respectively. Deposition time: 120 s. The error bars refer to the standard deviation (n = 3) (the solid line refers to cadmium, and the dashed line refers to lead). The supporting electrolyte was a 0.05 mol L^−1^ acetate buffer (pH 4.6).

The effect of deposition potential on the peak currents of cadmium and lead was investigated in the range from −0.8 to −1.2 V. The accumulation time was 60 s. The concentrations of Cd(II) and Pb(II) were 1 × 10^−7^ and 5 × 10^−8^ mol L^−1^. The results obtained during this study are presented in Figure 5. A similar behavior was observed for the cadmium and lead peak currents, with an increase in the height of the analytical signal in the range from −0.8 to −0.9 V, followed by a decrease in the peak currents at more negative potential values. Further research was conducted at the deposition potential of −0.9 V.

**Figure 6:**
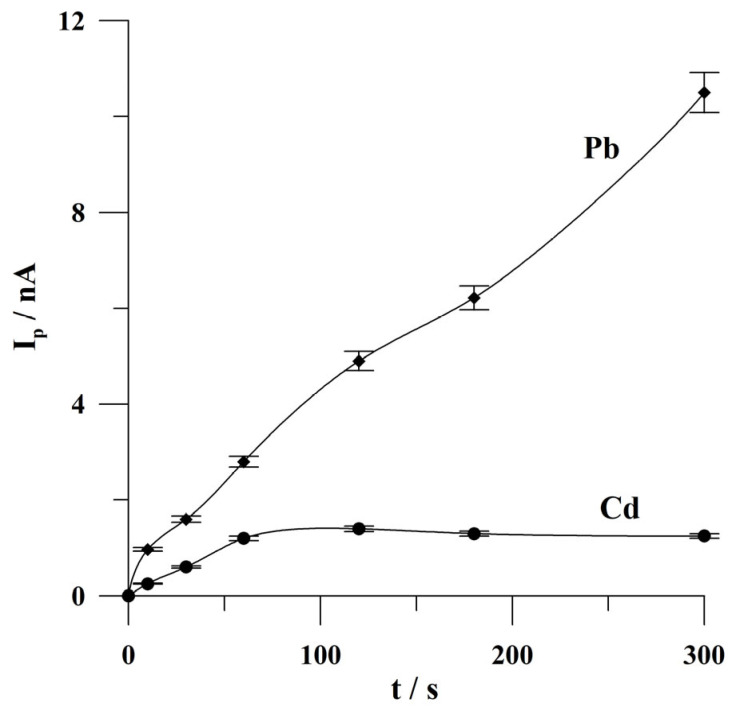
The effect of deposition time on the peak currents of Cd and Pb. The concentrations of Cd(II) and Pb(II): 2 × 10^−8^ mol L^−1^ and 1 × 10^−8^ mol L^−1^, respectively. Deposition potential: −0.9 V. The error bars refer to the standard deviation (n = 3). The supporting electrolyte was a 0.05 mol L^−1^ acetate buffer (pH 4.6).

The effect of deposition time on the peak currents of Cd(II) and Pb(II) was examined from 10 to 300 s. The concentrations of Cd(II) and Pb(II) were 2 × 10^−8^ and 1 × 10^−8^ mol L^−1^, respectively. When examining the effect of deposition time, lower concentrations of Cd(II) and Pb(II) were taken to avoid the saturation of the electrode’s surface at short deposition times. The results obtained are shown in Figure 6. It was found that the lead peak current increased almost linearly over the entire tested time range, while the cadmium peak current increased linearly from 10 to 60 s, and then no change in the cadmium analytical signal was observed. Further research was conducted at 60 s of deposition.

### 2.5. The Optimization of Square Wave Parameters

The next step of the measurements was optimizing the square wave parameters (step potential, amplitude, and frequency). During these studies the concentrations of Cd(II) and Pb(II) were 1 × 10^−7^ and 5 × 10^−8^ mol L^−1^, respectively.

The effect of step potential on Cd(II) and Pb(II) peak currents was studied from 2 to 8 mV, while frequency and amplitude were 200 Hz and 20 mV, respectively. Cd(II) and Pb(II) analytical signals increased in the range from 2 to 6 mV, obtained the highest value at 6 mV, and then decreased at a step potential of 8 mV. The value of the step potential of 6 mV was chosen for further studies.

The effect of amplitude on Cd(II) and Pb(II) peak currents was examined from 10 to 60 mV, while the frequency and step potential were 200 Hz and 6 mV, respectively. The Cd(II) and Pb(II) signals increased with increasing amplitude values up to 20 mV and reached the highest and constant peak currents within the range of 20 to 60 mV. Furthermore, it was observed that the most favorable S/B (signal-to-background) ratio was obtained at an amplitude of 20 mV for cadmium ions and an amplitude range of 10 to 20 mV for lead ions. Taking into account these results, further studies were conducted at an amplitude of 20 mV.

The effect of frequency on Cd(II) and Pb(II) peak currents was examined from 10 to 250 Hz. The amplitude and step potential were 20 and 6 mV, respectively. It was found that Cd(II) and Pb(II) peak heights increased in the whole range of tested frequency values from 10 to 250 Hz. Moreover, the calculated values of the S/B ratio indicated that the most favorable conditions for Cd(II) determination were observed at a frequency of 200 Hz, while for Pb(II) determination, they were observed in the range from 200 to 250 Hz. Based on these results, further research was conducted at a frequency of 200 Hz.

### 2.6. Calibration Data

The calibration studies were performed via sequential additions of increasing concentrations of Cd(II) and Pb(II) to the electrochemical vessel. Analytical parameters were calculated from the five-repetition series. The calibration graph for Cd(II) determination for the deposition time of 60 s was linear in the range of 5 × 10^−9^ to 2 × 10^−7^ mol L^−1^ and following the equation y = 0.12x − 0.88, where y and x are the peak current in nA and the Cd(II) concentration in nmol L^−1^, respectively. The linear correlation coefficient r was 0.994. The relative standard deviation (RSD) from five determinations of cadmium at a concentration of 5 × 10^−8^ mol L^−1^ was 4.8%. The calibration graph for Pb(II) determination at a deposition time of 60 s was linear in the range of 2 × 10^−9^ to 2 × 10^−7^ mol L^−1^ and followed the equation y = 0.23x − 0.06. The linear correlation coefficient r was 0.995. The RSD from five determinations of lead ions at a concentration of 2 × 10^−8^ mol L^−1^ was 4.3%.

**Figure 7:**
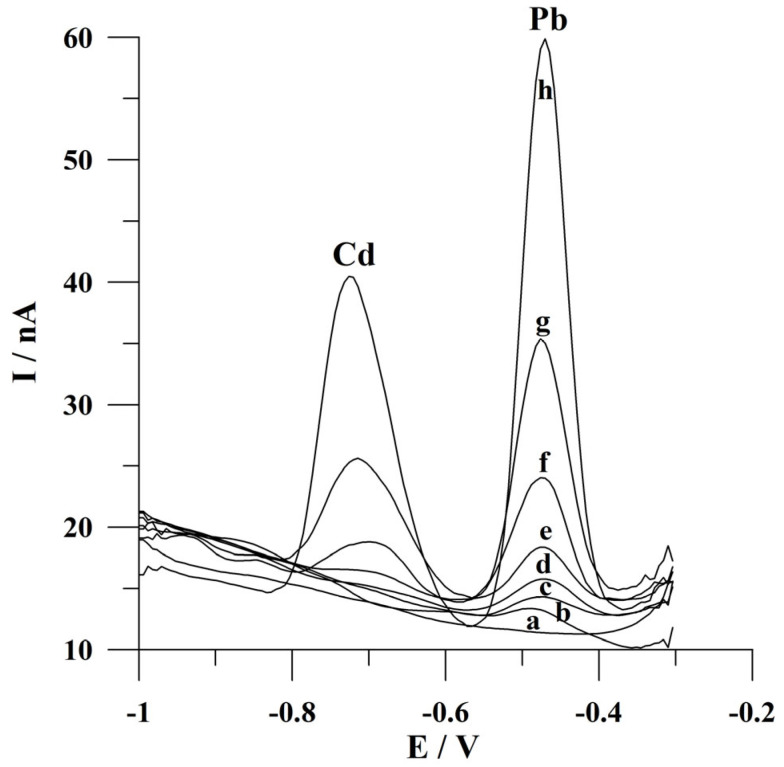
Square wave voltammograms obtained for increasing concentrations of Cd(II) and Pb(II): (a) 0; (b) 0 and 2 × 10^−9^; (c) 5 × 10^−9^ and 5 × 10^−9^; (d) 1 × 10^−8^ and 1 × 10^−8^; (e) 2 × 10^−8^ and 2 × 10^−8^; (f) 5 × 10^−8^ and 5 × 10^−8^; (g) 1 × 10^−7^ and 1 × 10^−7^; (h) 2 × 10^−7^ and 2 × 10^−7^ mol L^−1^. Potential and time of deposition: −0.9 V, 60 s. Frequency, amplitude, and step potential: 200 Hz, 20 mV, and 6 mV, respectively. The supporting electrolyte was a 0.05 mol L^−1^ acetate buffer (pH 4.6).

The detection limits for Cd(II) and Pb(II) following a deposition time of 60 s were calculated as 3 s m^−1^ (where s is a standard deviation for a low concentration of Cd(II) or Pb(II) and m is the slope of the calibration plot) and the values were 2.3 × 10^−9^ mol L^−1^ and 8.9 × 10^−10^ mol L^−1^. In the course of determining lead ions, a further decrease in the detection limit might be achieved by conducting the measurements at longer deposition times. Voltammograms obtained for increasing concentrations of Cd(II) and Pb(II) during calibration studies are presented in Figure 7. A comparison of the analytical parameters of the presented procedure with the previously reported voltammetric procedures using various working microelectrodes for Cd(II) and/or Pb(II) determination is summarized in Table 1. In addition, the application of a working microelectrode with high durability and attractive ecological properties is an undoubted advantage of the presented solution.

### 2.7. Repeatability Studies

The repeatability of the analytical signals obtained at the solid bismuth microelectrode array used for simultaneous Cd(II) and Pb(II) determination was investigated. The repeatability calculated for the data obtained within one day was examined based on the heights of the analytical signals of Cd and Pb recorded seven times from the same solution containing Cd(II) and Pb(II) ions at concentrations of 1 × 10^−7^ mol L^−1^ and 5 × 10^−8^ mol L^−1^, respectively. The obtained RSD values were equal to 4.3% and 3.9% for cadmium and lead, respectively. The repeatability calculated for the data obtained between different days was examined from different solutions containing 1 × 10^−7^ mol L^−1^ and 5 × 10^−8^ mol L^−1^ of Cd(II) and Pb(II), respectively. The obtained RSD values were equal to 5.1% and 4.9% for cadmium and lead, respectively. To summarize, it can be concluded that the analytical procedure demonstrates a satisfactory level of repeatability.

### 2.8. Interference Studies

The interference effects of foreign ions were studied for a solution containing Cd(II) and Pb(II) ions at a concentration of 5 × 10^−8^ mol L^−1^. The deposition time was 60 s. The influence of the foreign ions added on the Cd(II) and Pb(II) peak currents is shown in Table 2. In the presence of a large excess of other metal ions, a decrease in Cd and Pb signals was observed; therefore, the standard addition method is recommended for use. It should be noted that in most procedures for the voltammetric determination of these ions using bismuth electrodes, more negative deposition potential values have been used, and a greater influence of foreign ions has been found when reducing to the metallic state at more negative potential values.

### 2.9. Analytical Application

**Figure 8:**
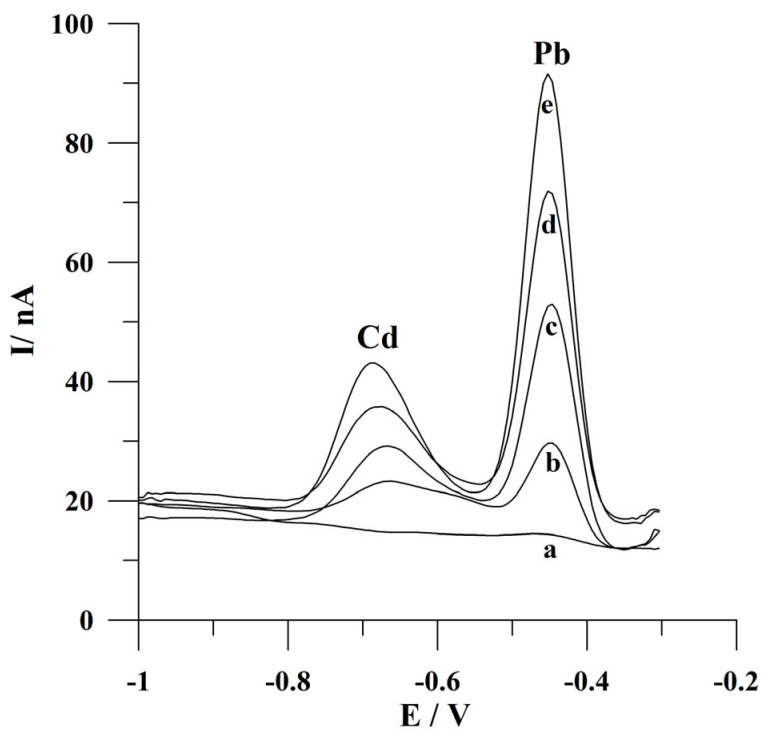
Square wave voltammograms obtained during the analysis of the certified water reference material TM 26.5: (a) blank; (b) diluted sample of reference material; (c) as (b) + 4 × 10^−8^ mol L^−1^ Cd(II) and 3 × 10^−8^ mol L^−1^ Pb(II); (d) as (b) + 8 × 10^−8^ mol L^−1^ Cd(II) and 6 × 10^−8^ mol L^−1^ Pb(II); (e) as (b) + 1.2 × 10^−7^ mol L^−1^ Cd(II) and 9 × 10^−8^ mol L^−1^ Pb(II). Deposition conditions: −0.9 V and 60 s. Frequency, amplitude, and step potential: 200 Hz, 20 mV, and 6 mV, respectively.

The developed voltammetric procedure was applied for the analysis of water-certified reference material TM 26.5 (water from Lake Ontario). Before a voltammetric analysis, the reference material was diluted (factor of dilution 1.56) and neutralized with an appropriate addition of 2 mol L^−1^ NaOH. The determinations were carried out using the standard additions method following a deposition time of 60 s. The declared cadmium and lead ion contents in this material are 7.11 ± 0.45 µg L^−1^ and 10.1 ± 0.8 µg L^−1^, respectively. The obtained concentrations of Cd(II) and Pb(II) in the certified reference material following the developed procedure were equal to 6.77 µg L^−1^ with a relative standard deviation of 4.7% (n = 5) and 9.88 µg L^−1^ and a relative standard deviation of 4.4% (n = 5), respectively. The obtained concentrations are consistent with the certified values and indicate the possibility of Cd(II) and Pb(II) determination in environmental water samples following the presented voltammetric procedure. The voltammograms obtained in the course of determining ions of interest in certified reference material are presented in Figure 8.

**Figure 9:**
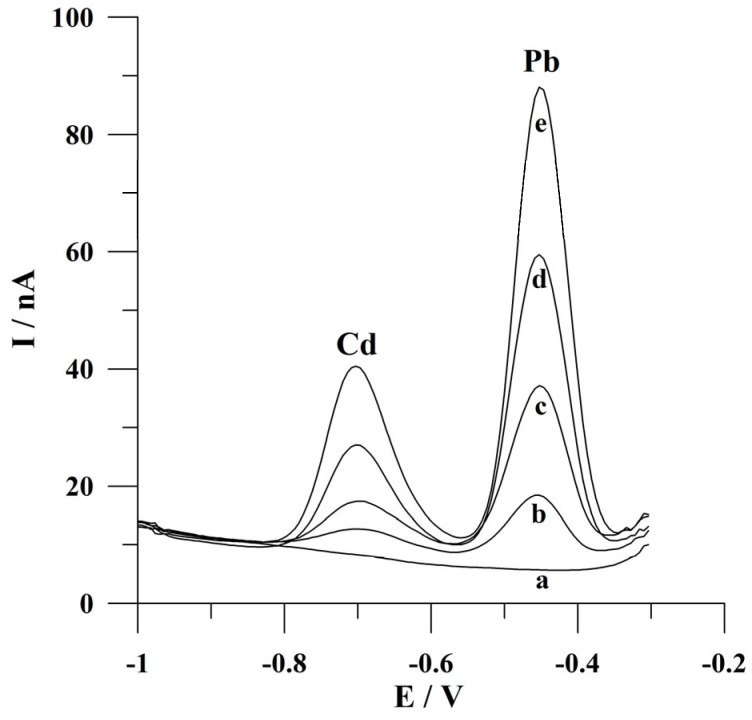
Square wave voltammograms obtained during the analysis of the natural water sample: (a) diluted water sample; (b) as (a) + 5 × 10^−8^ mol L^−1^; (c) as (a) + 1 × 10^−7^ mol L^−1^; (d) as (a) + 1.5 × 10^−7^ mol L^−1^; (e) as (a) + 2 × 10^−7^ mol L^−1^. Deposition conditions: −0.9 V and 60 s. Frequency, amplitude, and step potential: 200 Hz, 20 mV, and 6 mV, respectively.

Furthermore, the presented voltammetric procedure was applied for Cd(II) and Pb(II) determination in an environmental water sample from the Bystrzyca River (factor of dilution 2). The preparation of the sample is described in detail in the Experimental section. The measurements were carried out using the standard addition method. The possible Cd(II) and Pb(II) concentration levels were below the detection limit of the developed procedure, which was concluded by the absence of cadmium and lead peak currents on voltammograms recorded from the Bystrzyca River water. Because of this fact, recovery studies were performed by spiking the samples analyzed with fixed concentrations of Cd(II) and Pb(II). The samples were analyzed at a deposition time of 60 s. The average recovery values were in the range of 93 to 103% with a relative standard deviation (RSD) of 4.7% (n = 5) and 94 to 104% with a relative standard deviation of 4.5%, respectively, for cadmium and lead. The voltammograms obtained during the analysis of a natural water sample are presented in Figure 9. The obtained recovery values confirmed that the presented voltammetric procedure can be used for Cd(II) and Pb(II) determination in real water samples.

## 3. Materials and Methods

### 3.1. Instrumentation

The µAutolab analyzer (Eco Chemie, Utrecht, The Netherlands) was used to conduct the voltammetric measurements. A three-electrode electrochemical cell at a volume of 10 mL was used. A solid bismuth microelectrode array, consisting of forty-three single chromatographic capillaries of an inner diameter of about 10 µm filled with metallic bismuth and packed in one casing, was used as a working electrode. The construction of a working electrode was conducted as follows. To prepare a solid bismuth microelectrode array, ten chromatographic capillaries at a length of about 25 cm, an outer diameter of 140 μm and an inner diameter of 10 μm were filled with metallic bismuth according to the procedure described in [44]. Shortly, capillaries were inserted into the quartz tube with an inner diameter of 2 mm. Spaces between the capillaries and the quartz tube were sealed with epoxy resin at one end. To fill the capillaries with bismuth, metallic bismuth was placed in the quartz tube with an inner diameter of 5 mm and a length of 20 cm. Next, capillaries in the quartz tube were placed in a tube containing bismuth. The tube containing bismuth and capillaries was transferred to the furnace and heated at a temperature of 600 °C. During heating, 5 cm of the tube was outside the furnace. Within about 10 min, the bismuth melted, and a vacuum was connected to the capillaries in the quartz tube. Under these conditions, bismuth was forced into the capillaries. Next, the capillaries filled with bismuth were cut into sections at a length of 5 cm. Forty-three of them were packed in a quartz tube containing epoxy resin Araldite F mixed with hardener HY 905. Then, the tube containing capillaries and resin was heated at 110 °C within 36 h. The obtained array of capillaries was cut into sections with a length of 5 mm. Such a prepared array was inserted into PEEK housing. Electrical contact from the array was obtained using carbon and brass wire. According to this procedure, eight microelectrode arrays were prepared. Five of them were used for the studies presented below. The design of the microelectrodes array described in this article resulted in the attainment of a long-term operational and highly stable working electrode. The proposed sensor can be used for a minimum period of 12 months, which was confirmed by the absence of substantial alterations in the analytical signals of ions of interest, as evidenced by studies conducted over the designated timeframe. The electrode was prepared for the measurements by polishing it with 2500-grit sandpaper (Matador APP, Poland), which was conducted immediately prior to the commencement of the measurement series and before each analysis of a real sample. After polishing, the microelectrode array was sonicated in deionized water using an ultrasonic cleaner (Sonic-3, Polsonic, Poland) for about 30 s. Prior to each measurement, the microelectrode surface was prepared by applying an activation step, which was incorporated into the standard measurement procedure to reduce bismuth oxides that can be formed on the microelectrode’s surface in the presence of oxygen dissolved in the solution used in the studies. The parameters of this procedure were optimized and described in Section 2.3. Ag/AgCl/NaCl_sat._ and platinum wire were used as reference and auxiliary electrodes, respectively.

### 3.2. Preparation of the Sample

A real water sample from the Bystrzyca River (eastern Poland) was stored in a refrigerator until use. Prior to the direct analysis, the water sample was filtered using a membrane filter (0.45 μm).

### 3.3. Reagents

A 1 mol L^−1^ acetate buffer CH_3_COOH/CH_3_COONa of pH 4.6 was prepared from TraceSELECT reagents purchased from Sigma Aldrich. The stock solutions of Cd(II) and Pb(II) at a concentration of 1 g L^−1^ were purchased from Fluka (Buchs, Switzerland). The working solutions of Cd(II) and Pb(II) at a concentration of 1 × 10^−5^ mol L^−1^ were prepared by diluting the stock solutions in 0.01 mol L^−1^ HNO_3_ as required. Other reagents were obtained from POCh (Gliwice, Poland) and were used as received. Water used to prepare all solutions was purified in the Milli-Q system purification. The lake water-certified reference material TM-26.5 (Lake Ontario water), used for the validation of the procedure, was purchased from Environment and Climate Change (Gatineau, QC, Canada).

### 3.4. The Standard Procedure of the Measurements

The analyzed sample was pipetted into the voltammetric cell. Then, 0.5 mL of a 1 mol L^−1^ acetate buffer (pH 4.6) was added and made up to a volume of 10 mL with deionized water. The anodic stripping voltammetric measurements consisted of the following steps. Firstly, the activation step was carried out by applying the working microelectrode array with a short potential pulse of −2.5 V within 1 s. Secondly, the deposition time was conducted at −0.9 V within 60 s. During this step, cadmium and lead ions underwent reduction to a metallic state on the surface of the working electrode. Then, after a ten-second equilibration step, a square wave voltammogram was registered while the potential was changed from −1.0 to −0.3 V. The frequency, amplitude, and step potential were 200 Hz, 20 mV, and 6 mV, respectively. The research was conducted without the solution deoxygenation. Unless otherwise stated, each measurement was repeated three times.

## 4. Conclusions

In the present article, a novel method of constructing a solid metal microelectrode array is presented. The resulting sensor exhibits significant properties, including an extended service life, durability, environmental friendliness, and no need for surface modifications. Taking into account the stability and durability of the proposed solid bismuth microelectrode array and the fact that the surface does not require frequent polishing, it can potentially be applied for measurements in flow systems. Furthermore, thanks to the use of the proposed working microelectrode, the supporting electrolyte composition is uncomplicated, and the standard measurement procedure is relatively simple and short. The presented solid bismuth microelectrode array was utilized for the development of the voltammetric procedure and the simultaneous determination of Cd(II) and Pb(II) by anodic stripping voltammetry. The procedure presented is characterized by satisfying selectivity, good precision, and a wide linear dynamic range for the determination of both ions. The fact that the results obtained during the analysis of certified reference material correspond to the certified values confirms the correctness of the developed analytical procedure. Satisfactory recovery values during the analysis of the environmental water sample were also obtained.

## Figures and Tables

**Table 1:** The comparison of parameters used in analytical procedures for determining cadmium and lead ions using various working microelectrodes via stripping voltammetry.

Working Electrode	Linear Range [nmol L^−1^]		Detection Limit [nmol L^−1^]		Ref.
	Cd(II)	Pb(II)	Cd(II)	Pb(II)	
On-chip-generated HgµE	-	100–400	-	100	[26]
Vibrating Silver amalgam µE	-	-	0.079	0.008	[27]
Gold disk µE	1.8–180	0.97–145	-	-	[28]
Bi/carbon fiber µE	50–350	50–350	9.2	10	[29]
Nitrogen-doped diamond-like carbon µE array	10–120	10–120	2.9	2.4	[33]
Hg/diamond-like carbon µE array	16–83	16–83	-	-	[34]
Solid Bi µE array	5–200	2–200	2.3	0.89	[this work]

Abbreviations: µE—microelectrode.

**Table 2:** Relative Cd(II) and Pb(II) analytical signals in the presence and absence of foreign ions. The Cd(II) and Pb(II) concentration was 5 × 10^−8^ mol L^−1^. Deposition conditions: −0.9 V and 60 s.

Foreign Ion	Molar Excess of Foreign Ion	* Relative Signal of Cd(II)	* Relative Signal of Pb(II)
V(V)	100	0.83	1.04
Ni(II)	100	0.54	0.81
Co(II)	100	0.84	0.86
Fe(III)	100	0.75	0.91
Mn(II)	100	0.73	0.87
Zn(II)	100	0.85	0.99
Mo(VI)	10	0.84	0.95
	20	0.22	0.38
Cu(II)	10	0.21	0.35
	20	0.06	0.27

* Relative signal—the peak current ratio in the presence and absence of a molar excess of foreign ions.

## Data Availability

The data are contained within the article.
